# Factors associated with postmenstrual age at full oral feeding in very preterm infants

**DOI:** 10.1371/journal.pone.0241769

**Published:** 2020-11-11

**Authors:** Grégoire Brun, Céline J. Fischer Fumeaux, Eric Giannoni, Myriam Bickle Graz

**Affiliations:** 1 Emergency Care, Lausanne University Hospital and University of Lausanne, Lausanne, Switzerland; 2 Clinic of Neonatology, Department Mother-Woman-Child, Lausanne University Hospital and University of Lausanne, Lausanne, Switzerland; 3 Follow-up Unit, Department Mother-Woman-Child, Lausanne University Hospital and University of Lausanne, Lausanne, Switzerland; Centre Hospitalier Universitaire Vaudois, FRANCE

## Abstract

**Aim:**

We aimed to identify variables associated with gestational age at full oral feeding in a cohort of very preterm infants.

**Methods:**

In this retrospective study, all infants born below 32 weeks of gestation and admitted to a level III neonatal unit in 2015 were included. We dichotomized our population of 122 infants through the median age at full oral feeding, and explored which variables were statistically different between the two groups. We then used linear regression analysis to study the association between variables known from the literature and variables we had identified and age at full oral feeding.

**Results:**

The median postnatal age at full oral feeding was 36 6/7weeks post menstrual age (Q1-Q3 35 6/7-392/7), and was associated with the duration of hospital of stay. In the univariable linear regression, the variables significantly associated with full oral feeding were gestational age, socioeconomic status, sepsis, patent ductus arteriosus, duration of supplementary oxygen, of non-invasive and invasive ventilation, and bronchopulmonary dysplasia. In the multivariable regression analysis, duration of non-invasive ventilation and oxygen therapy, bronchopulmonary dysplasia, and patent ductus arteriosus were associated with an older age at full oral feeding, with bronchopulmonary dysplasia the single most potent predictor.

**Discussion:**

Lung disease severity is a major determinant of age at full oral feeding and thus length of stay in this population. Other factors associated with FOF include socioeconomic status and patent ductus arteriosus, There is a need for research addressing evidence-based bundles of care for these infants at risk of long-lasting feeding and neurodevelopmental impairments.

## Introduction

Worldwide, 10% of children are born preterm [1] and are at risk of lifelong disabilities, including neurodevelopmental impairments. Brain growth and maturation is of paramount importance during these last weeks of gestation, and depends crucially on adequate nutrition [2]. Very preterm neonates generally require parenteral nutrition, while enteral feeding is provided in increasing doses. Once full enteral feeding is achieved, the transition period from tube to full oral feeds (FOF) is a major determinant of the length of stay in the neonatal unit [3] and depends notably on the maturation and efficiency of the coordination of sucking-swallowing and breathing. Prolonged tube feeding is associated with long-term feeding difficulties [4], long-term oral sensitivity and speech problems [5]. Finally, feeding difficulties may have a negative impact on nutrition in the neonatal period and thereafter, which may lead to stunted growth, altered neurodevelopment and lower academic achievement, and potentially adult onset metabolic disease [6]. Among the many complications of preterm birth, feeding and nutritional issues thus carry a heavy burden in terms of financial and social cost [7].

Different factors are known to be associated with age at tube weaning, such as gestational age, need for respiratory support and bronchopulmonary dysplasia (BPD) [8], as well as parental involvement [3]. The association of socio-economic status with several neurodevelopmental outcomes in preterm children has been demonstrated [9], but the association with feeding is underreported [10, 11]. Moreover, studies of oral motor interventions to enhance feeding skills, such as non-nutritive sucking and oral stimulation, do not show unanimous results [12–15], which could be explained by the heterogeneity of the populations and of the interventions.

The aim of this study was thus to identify and characterize, in infants born before 32 weeks of gestation, the variables associated with postmenstrual age (PMA) at full oral feeding.

## Patients and methods

The "Commission cantonale d'éthique de la recherche sur l'être humain" approved the study protocol, and all patients gave written consent.

This was a retrospective cohort study of infants born before 32 weeks of gestation and admitted to a level III neonatal unit admitting 800 infants per year, as the reference center for a region counting 15 000 births/year [16]. All infants born between 01.01.2015 and 31.12.2015 at a gestational age less than 32 weeks were considered for this study. Parents were asked for written informed consent for the use of neonatal and follow-up data, according to the Swiss regulation and local ethics committee. Exclusion criteria were consent refusal, death before full oral feeding, and major congenital abnormalities.

### Feeding practice

In the setting of our neonatal unit in 2015, enteral feeds were started on the first day of life with mother’s own milk when available, fresh or frozen, or preterm formula (BEBA Alprem or BEBA Aliment pour Prématurés Etape 1; Nestlé, Vevey, Switzerland). The milk was administered every 2 hours for preterm infants weighing < 1500g and every 3 hours for those weighing ≥ 1500g, with a targeted average daily volume increase of 10–20 ml/kg/day, adapted according to enteral tolerance until 160 ml/kg/day. Breastfeeding was encouraged as early as possible, with no lower age limit, whereas bottle-feeding was started from 34 weeks PMA. Donor milk was not available. A standard fortifier (Aptamil Frauen-Milch-Supplement 4%; Milupa SA, Domdidier, Switzerland) was introduced when 100 mL/kg/day of human milk was tolerated for all preterm infants < 32 weeks of GA. To assess feeding tolerance, gastric residuals were checked before every feed as a part of enteral feeding indicator; when the volumes of aspirates were superior to volumes of feeds, and/or in the presence of severe abdominal distension, it was possible to suspend enteral feeding for a couple of hours until resolution of symptoms.

Probiotics were not administered in 2015, due to concerns regarding safety of available products [17]. Oral stimulation and non-nutritive sucking were systematically offered to all preterm infants as part of general developmental care, following the procedures described in Pfister et al [18], which include the use of pacifiers, exposure to the smell and taste of milk, massage, and stimulation of the rooting reflex. Parental presence and skin-to-skin practice were also strongly encouraged, but these interventions were not monitored. The feeding tube was removed when the infant was able to breast or bottle-feed more than 60% of their total milk intake.

### Variables

Neonatal physiological and treatment variables were prospectively recorded in a widely available computerized patient Clinical Information System (Metavision® iMDsoft, Massachussetts, USA) [19, 20], which allows the extraction of selected data. Social and diagnostic variables were prospectively recorded in a specific *ad hoc* database. The selection of independent variables for this study was based on the literature and on an exploratory analysis of our population. Variables used for the study were thus: gestational age, assessed with early first trimester ultrasound (before 14 weeks) and last menstrual period, gender, birthweight and birthweight z-score [21], small for gestational age (SGA) defined as birthweight z-score < 2, mode of delivery. Socioeconomic status was assessed with the score described by Largo [22], which entails mother’s education (scored from 1 to 6) and father’s occupation (scored from 1 to 6), which is used in neonatal research in Switzerland [23] (total score from 2 to 12, 2 corresponding to higher parental education or occupation). Blood culture proven sepsis [24], necrotising enterocolitis (Bell stage ≥2), major brain injury such as cystic leukomalacia or grade III-IV intraventricular haemorrhage according to Papile [25], In 2015, cardiac echocardiography to detect PDA was made on the base of suggestive clinical signs. Hemodynamically significant PDA was treated medically first (indomethacin) if there were no contraindications, surgically in the case of a failure of the medical treatment or in the presence of contraindications. Variables were thus treated PDA, including both medically and surgically treated PDA, as usually reported, and surgically treated PDA.

To assess the impact of lung disease, we used the diagnoses of bronchopulmonary dysplasia, defined as a requirement for more than 28 days of supplemental oxygen between birth and 36 weeks PMA [26], duration of invasive ventilation, of continuous positive airway pressure (CPAP), and supplementary oxygen corresponding to the total number of days for which the patient received more than 21% oxygen, whatever the mode of administration (low or high flow nasal cannula, non-invasive or invasive ventilation). Nasal CPAP using the Medijet® generator [27] was the primary mode of respiratory support in spontaneously breathing infants. Respiratory distress syndrome was managed according to the most recent guidelines [28], to ensure continuity of care between the different attending physicians. Finally studied outcomes were post menstrual age (PMA) at enteral and oral feeding milestones, type of milk at discharge (any or no mother’s own milk), and length of stay.

### Statistical analysis

Analyses were performed with STATA® 13 (StataCorp. 2013. Stata Statistical Software: Release 13. College Station, TX: StataCorp LP). Continuous variables were described with median and first to third quartiles, categorical variables with proportions. As a preliminary exploration, we divided our cohort in 2 subgroups on the basis of the median PMA at FOF (early PMA at FOF and late PMA). We tested the difference between the 2 groups for the above mentioned variables, with t-tests or Kruskall-Wallis test for continuous variables and chi2 test for categorical variables.

For the linear regression analysis, we chose variables which were significantly different in our preliminary exploration, as well as variables known from the literature to be associated with age at FOF. The association of the variables with PMA at FOF was analysed with unilinear regression first. Secondly, we used multilinear regression including variables which were significant at the p< 0.2 level in the univariable analysis.

## Results

**Fig 1:**
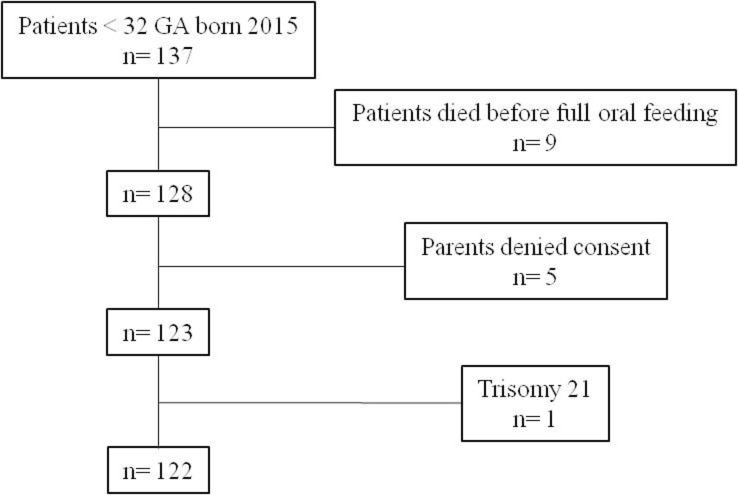
Flowchart of the study population.

The study population consisted of 122 patients (64 girls, 52%), with a median gestational age of 29.7 weeks (Q1-Q3 28–30.8, range 23 6/7/7-31 6/7) and a median birthweight of 1138 g (Q1-Q3 900–1425, range 510-1930g) (Fig 1), 88.5% of whom had received any antenatal steroids.

### Patient characteristics

Enteral feeding was initiated on the first day of life for most patients (n = 112, 9%). Weaning of the feeding tube occurred after a median of 55 days (Q1-Q3 38–74), at a median of 36 6/7 weeks PMA (Q1-Q3 356/7-39 2/7) with a wide range between 34 and 141 weeks. Patients were discharged home at a median postmenstrual age of 37 4/7 (Q1-Q3 36 3/7–40 3/7, range 34 4/7–109 weeks PMA). The age at discharge home was associated with age at FOF (coef. (IC95%) 4.06 (3.45; 4.67), p < 0.001). At discharge home 70% of infants were fed with some mother’s milk, and 30% with formula only.

The main patient characteristics are described in Table 1, with the characteristics of the groups of early (≤ median FOF) and late age at FOF (> median FOF).

**Table 1:** Population characteristics.

	All	Feeding tube weaned	Feeding tube weaned	P
	n = 122	≤ 36 6/7 weeks PMA	> 36 6/7 weeks PMA	
		n = 60	n = 62	
Socioeconomic status (median, Q1-Q3)	6 (4–7)	6 (4–7)	6 (4–7)	NS
Antenatal steroids (n, %)	108 (88.5)	54 (90)	54(87))	NS
Gestational age, weeks (median, Q1-Q3)	29.7 (28; 30.8)	30.4 (29.7; 31.4)	28.4 (27.4; 29.7)	0.001
Gender, female (n, %)	64 (52.4)	27 (45)	37 (59.7)	0.047
Birthweight (g, median, Q1-Q3)	1138 (900;1425)	1350 (1089; 1580)	1010 (770; 1155)	0.001
Birthweight z-score (mean, SD)	-0.38 (0.72)	-0.12 (0.64)	-0.55 (0.76)	0.007
SGA (z-score < -2DS, %)	3 (2.5)	0	3 (4.8)	NS
Caesarean section (%)	98 (80)	41 (68)	57 (92)	0.001
Blood culture-proven sepsis (%)	13 (10)	0 (0)	13 (21)	0.001
Necrotizing enterocolitis (%)	4 (3)	0 (0)	4 (6)	0.001
Treated patent ductus arteriosus (n, %)	22 (18)	6 (10)	16 (25.8)	0.011
Surgical PDA	6 (5)	1(1.7)	5(8.0)	NS
Major brain lesions^1^ (n, %)	10 (8.2)	2 (3.3)	8 (12.9)	0.048
Supplementary oxygen (days, median, Q1-Q3)	2 (0; 28)	0 (0; 1)	25 (5; 44)	0.0001
Range	0–284	0–45	0–284	
Duration of CPAP^2^ (days, median, Q1-Q3)	23 (6; 41)	7 (2; 19)	38 (26; 51)	0.0001
Range	0–130	0–55	0–130	
Duration of invasive ventilation (days, median, Q1-Q3)	0.5 (0; 2)	0.5 (0; 2)	1 (0; 6)	0.0001
Range	0–10	0–52	0–52	
Length of stay (days, median, Q1-Q3)	62 (42–86)	43 (36–51)	82 (66–107)	0.0001

p values were computed with student’s t-test or Kruskall-Wallis test for normally distributed and non-normally distributed continuous variables and with chi2 tests for categorical variables.

1: Major brain lesions: Cystic leucomalacia and/or grade III-IV intraventricular hemorrhage.

2: CPAP: continuous positive airway pressure.

### Univariable linear regression analyses

Variables significantly associated with PMA at FOF were BPD (+6 days), parental socioeconomic status, with a better status (i.e. lower score) associated with younger PMA at FOF as the maximum difference of 10 points in SES could add 11 days of FOF, gestational age, blood culture proven sepsis (+ 12 days) and treated PDA (+ 5.7 days), surgically treated PDA (+19 days) (Table 2). Respiratory support variables were strongly associated with the outcome, as each week of supplementary oxygen added 1.6 days of feeding tube, each week of CPAP 1.7 day of feeding tube and each day of invasive ventilation added 0.9 days of feeding tube. No association could be shown for gender, birthweight z-score, SGA, necrotizing enterocolitis, mode of delivery and major brain lesions with age at FOF.

**Table 2:** Univariable regression.

	Coefficient (CI 95%)	Beta coefficient	R^2^	P
**Socioeconomic status**	**1.13 (0.27 ; 2)**	**0.24**	**0.06**	**0.011**
**Gestational age (weeks)**	**-2.05 (-3.14; -0.95)**	**-0.32**	**0.1**	**<0.001**
Female gender	1.16 (-3.11 ; 5.44)	0.049	0.0024	0.59
Birthweight z-score	-1.28 (-4.25 ; 1.68)	-0.079	0.0061	0.394
SGA^1^	0.7 (-13.09 ; 14.49)	0.0091	0.0001	0.92
Vaginal delivery	-2.03 (-7.39 ; 3.33)	-0.068	0.0047	0.45
**Sepsis**	**12.64 (6.11; 19.17)**	**0.33**	**0.11**	**<0.001**
Necrotizing enterocolitis	10.73 (-1.1 ; 22.57)	0.16	0.026	0.075
**Treated PDA**	**5.75 (-0.45; 10.8)**	**0.19**	**0.036**	**0.036**
**Surgically treated PDA**	**19.24 (9.99; 28.48)**	**0.35**	**0.12**	**<0.001**
Major brain lesion	1.98 (-5.8 ; 9.75)	0.073	0.0021	0.616
Mother’s milk at discharge (any)	1.81 (-2.64; 6.27)	0.046	0.0054	0.422
**Oxygen treatment(days)**	**0.23 (0.18 ; 0.28)**	**0.67**	**0.44**	**<0.001**
**CPAP**^**3**^**(days)**	**0.24 (0.16 ; 0.32)**	**0.47**	**0.23**	**<0.001**
**Invasive ventilation (days)**	**0.92 (0.71 ; 1.13)**	**0.62**	**0.38**	**<0.001**
**Bronchopulmonary dysplasia**	**6.41 (1.65; 11.2)**	**0.23**	**0.05**	**0.008**

1. Small for gestational age

2. PDA, patent ductus arteriosus

3 CPAP: continuous positive airway pressure

### Multivariable linear regression analyses

The variables for the multivariable regression were socioeconomic status, gestational age, sepsis, necrotizing enterocolitis, treated PDA and surgically treated PDA and the 4 respiratory variables (supplementary oxygen and CPAP, invasive ventilation, and bronchopulmonary dysplasia).The final analysis is shown in Table 3.

**Table 3:** Multivariable linear regression.

PMA at full oral feeding	Coefficient (CI 95%)	P
Socioeconomic status	0.29 (-0.24 , 0.82)	0.282
Gestational age (days)	0.85 (-0.26; 1.96)	0.133
Sepsis	2.02 (-3.74; 7.78)	0.488
Necrotizing enterocolitis	-4.00 (-13.8.; 5.81)	0.420
Treated patent ductus arteriosus	-5.60 (-9.82; -1.38)	**0.010**
Surgically treated PDA	6.52 (-0.83; 13.8)	0.082
Invasive ventilation (days)	0.36 (-0.11 ; 0.73)	0.058
CPAP^1^ (days)	0.12 (0.01; 0.23)	**0.032**
Oxygen treatment (days)	0.23 (0.14 ; 0.32)	**<0.001**
Bronchopulmonary dysplasia	-11.23 (-15.5; -6.9)	**<0.001**

In the multivariable linear regression (p< 0.0001, r^2^ 0.67), bronchopulmonary dysplasia was the most important single predictor of delayed FOF, followed by oxygen and CPAP duration, as well as treated PDA (any treatment).

PMID: 30974433

## Discussion

This study examined the association of main neonatal characteristics with the postmenstrual age at which our cohort of very preterm infants were weaned from the feeding tube. FOF was achieved at a median of 36 6/7 weeks PMA (Q1-Q3 35 6/7-39 2/7) within the upper reported range of 35 1/7–36 6/7 weeks PMA [8, 29, 30].

The univariable regression showed a weak but significant association of socioeconomic status, gestational age, sepsis and PDA with the outcome, whereas all variables linked with respiratory support (supplementary oxygen, duration of CPAP, of invasive ventilation, and, BPD) and surgically treated PDA were associated with the outcome with moderate to large effect sizes. Not surprisingly, the multivariable analysis also showed that supplementary oxygen, CPAP, invasive ventilation, BPD and PDA were associated with PMA at FOF. BPD, which affected 25% of our population, was the single most potent predictor, followed by treated PDA.

Our findings are similar to other studies for some variables such as gestational age [29], with each supplementary week decreasing the age at FOF by 2 days, or treated patent ductus arteriosus [8, 30, 31],. The mechanism through which PDA is linked with FOF is not clear, PDA may be seen as a marker of the severity of the infant’s condition. Contrary to most published studies, there was no association with SGA, which could be explained by the varying definition of SGA, based on a birthweight z score <-2 SD in our cohort, which included very few patients (3%), which were all in the late FOF group, nor with NEC, which was a rare event in our cohort.

Better socio-economic status was associated with earlier FOF in the univariable model, although this result needs to be interpreted with caution, as multiple testing may impact the significance of some results. To our knowledge, this association has not been studied before, but the association of socioeconomic status with parental presence and skin to skin care [32], and breastfeeding during NICU [11, 33], could have mediated this outcome. The association of socio-economic status with later feeding difficulties in preterm children has also been shown [34].

Respiratory support was a risk factor for delayed FOF in several studies [8, 29–31, 35] with several possible explanations. In most neonatal units, infants are not offered oral feeds as long as they are treated for respiratory distress (mainly by fear of aspiration). The transition period from tube to oral feeding thus starts later for these infants. Moreover, infants treated by invasive or non-invasive ventilation experience nociceptive stimuli in the naso- and orofacial region, which can lead to altered processing of sensory information [36], as well as with olfactory stimuli that are known to be very important in feeding processes particularly at this age. Moreover, in a population of very preterm infants, Neubauer showed that BPD and delayed FOF were independently associated with delayed brain maturation at term-equivalent age [37]. Delayed age at FOF may thus be seen as an early marker of immature sensorimotor development and delayed or altered brain maturation, a risk factor for long-lasting neurodevelopmental impairments [38–40].

The major strengths of this study are the prospective collection of data entered and retrieved from our clinical information system Metavision**®** and in our study database. The analysis of respiratory support was based on objective measures of duration of support, as well as the diagnoses of BPD, more subject to interpretation, [26].

The major limitation of this study is its single-centre retrospective design, with some variables of interest not available, such as time of parental presence, quantitative assessment of skin to skin care or oral stimulation which have been shown to be associated with FOF [3]. Other obstacles described by Tubbs- Cooley, the “missed opportunities”, where infants are tube fed for convenience, thus depriving them of feeding stimulations, were not recorded either [41]. The feeding protocol differed slightly between infants fed with mother’s milk, who could breastfeed as early as possible, and infants fed formula (30% at discharge), who were offered bottle feeding from 34 weeks on. The data about timing of these milestones, and about the moment mothers switched from breast to bottle was not recorded in our data set, so the impact of these variables was not analyzed. However, the type of milk at discharge was not associated with FOF. Finally, the risk of collinearity is high among the studied variables, such as the variables based on respiratory support, PDA, and gestational age, which warrants caution in the interpretation of the results.

### Perspectives

The feeding protocol has changed in our unit since this period and infants are offered a bundle of care including administration of oropharyngeal colostrum even for mechanically ventilated neonates, breastfeeding peer support, early bottle feeding when needed, systematic skin-to-skin care, and oral feeding under CPAP. The impact of these interventions need to be monitored. Recent studies also seem to show benefits in starting cue-based feeding preterm infants at a very early age [42]. Research on oral stimulation in unselected populations of preterm infants have shown inconclusive results according to a recent Cochrane review [14], due to methodological flaws such as varying inclusion criteria. We suggest that future studies of bundles including oral and olfactory stimulation, parental education and enhanced skin to skin care should target this specific group of infants with severe lung disease at risk of long-lasting feeding and neurodevelopmental difficulties.

## Conclusion

Lung disease was a major independent determinant of delayed feeding skills in this cohort of very preterm infants, whereas gestational age, socio-economic status, and sepsis, were associated with age at FOF in an univariable linear analysis only. These findings which replicate studies in different contexts, ought to guide research on interventions aimed at shortening this learning period to FOF and thus the length of stay.

## Supporting information

S1 Data (XLSX)

## Abbreviations

BPD: Bronchopulmonary dysplasia
CPAP: continuous positive airway pressure
FOF: Full oral feeding
PMA: post menstrual age
SGA: small for gestational age
